# 5-*tert*-Butyl 1-ethyl 3-amino-1,4,5,6-tetra­hydro­pyrrolo­[3,4-*c*]pyrazole-1,5-dicarboxyl­ate

**DOI:** 10.1107/S1600536811013444

**Published:** 2011-04-16

**Authors:** Wen-Bin Xia, Xiao-Guang Bai, Hong-Tao Liu, Ju-Xian Wang

**Affiliations:** a School of Chemical Engineering and Technology, Tianjin University, Tianjin 300072, People’s Republic of China; b Institute of Medicinal Biotechnology, Chinese Academy of Medical Sciences, and Peking Union Medical College, Beijing 100050, People’s Republic of China

## Abstract

The asymmetric unit of the title compound, C_13_H_20_N_4_O_4_, contains two crystallographically independent mol­ecules in which the dihedral angles between the fused pyrrole and pyrazole rings are 5.06 (8) and 1.12 (8)°. In the crystal, mol­ecules are linked by inter­molecular N—H⋯O and N—H⋯N hydrogen bonds into chains parallel to the *b* axis.

## Related literature

For general background to potential anti­cancer kinase inhibitors, see: Fancelli *et al.* (2005 ▶); Gadekar *et al.* (1968 ▶). For the structure of a related compound synthesized by our group, see: Guo *et al.* (2010 ▶).
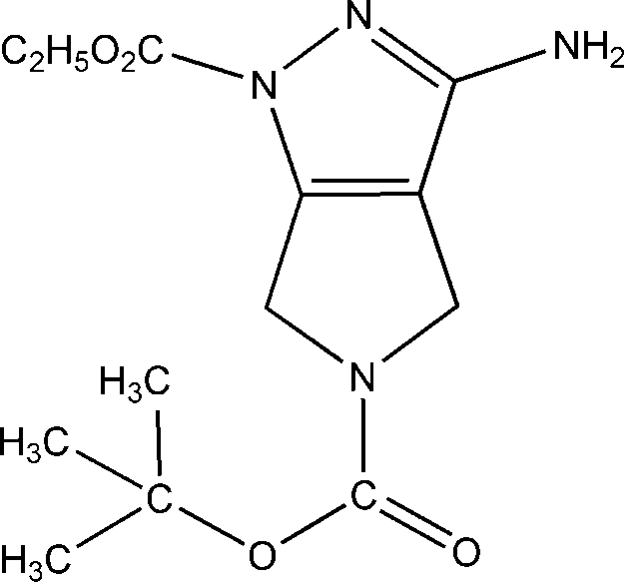


## Experimental

### 

#### Crystal data

C_13_H_20_N_4_O_4_
*M* *_r_* = 296.33
Triclinic, 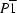

*a* = 10.772 (3) Å
*b* = 12.180 (4) Å
*c* = 12.986 (4) Å
α = 70.845 (5)°
β = 65.875 (4)°
γ = 85.821 (5)°
*V* = 1465.2 (7) Å^3^
*Z* = 4
Mo *K*α radiation
μ = 0.10 mm^−1^
*T* = 296 K
0.06 × 0.05 × 0.04 mm

#### Data collection

Bruker APEXII CCD diffractometer
Absorption correction: multi-scan (*SADABS*; Bruker, 2008 ▶) *T*_min_ = 0.994, *T*_max_ = 0.996
7444 measured reflections
5102 independent reflections
3915 reflections with *I* > 2σ(*I*)
*R*_int_ = 0.026

#### Refinement

*R*[*F* ^2^ > 2σ(*F* ^2^)] = 0.047
*wR*(*F* ^2^) = 0.128
*S* = 1.03
5102 reflections
387 parameters
H-atom parameters constrained
Δρ_max_ = 0.26 e Å^−3^
Δρ_min_ = −0.32 e Å^−3^

### 

Data collection: *APEX2* (Bruker, 2008 ▶); cell refinement: *SAINT* (Bruker, 2008 ▶); data reduction: *SAINT*; program(s) used to solve structure: *SHELXS97* (Sheldrick, 2008 ▶); program(s) used to refine structure: *SHELXL97* (Sheldrick, 2008 ▶); molecular graphics: *SHELXTL* (Sheldrick, 2008 ▶); software used to prepare material for publication: *SHELXTL*.

## Supplementary Material

Crystal structure: contains datablocks I, global. DOI: 10.1107/S1600536811013444/rz2580sup1.cif

Structure factors: contains datablocks I. DOI: 10.1107/S1600536811013444/rz2580Isup2.hkl

Additional supplementary materials:  crystallographic information; 3D view; checkCIF report

## Figures and Tables

**Table 1:** Hydrogen-bond geometry (Å, °)

*D*—H⋯*A*	*D*—H	H⋯*A*	*D*⋯*A*	*D*—H⋯*A*
N8—H8"⋯O4^i^	0.86	2.25	3.069 (3)	160
N8—H8′⋯N2^ii^	0.86	2.26	3.087 (3)	163
N4—H4′⋯N6^iii^	0.90	2.25	3.104 (3)	157
N4—H4"⋯O8^iv^	0.90	2.36	3.233 (3)	163

Symmetry codes: (i) 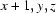; (ii) 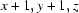; (iii) 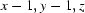; (iv) 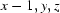.

**Fig. 1.**
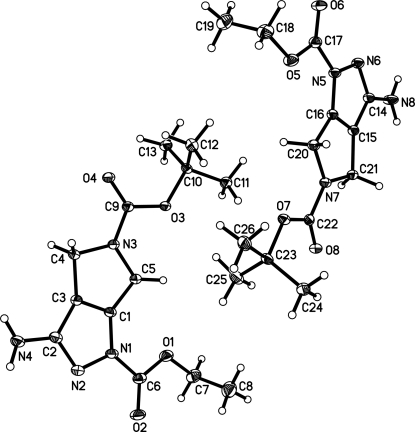
The molecular structure of the title compound with displacement ellipsoids drawn at the 30% probability level.

**Fig. 2.**
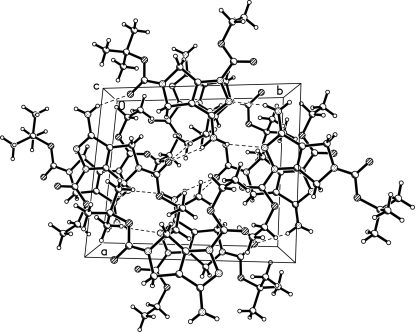
Packing diagram of the title compound viewed along the c axis. Hydrogen bonds are shown as dashed lines.

## supplementary crystallographic information

### Comment

As an extension of our work (Guo *et al.*, 2010) on the development of potential anticancer kinase inhibitors (Fancelli *et al.*, 2005; Gadekar *et al.*, 1968), we have synthesized the title compound and report its crystal structure herein.

The asymmetric unit of the title compound (Fig. 1) contains two crystallographically independent molecules. All bond lengths and angles have normal values. The dihedral angles between the fused pyrrole and pyrazole rings are 5.06 (8) and 1.12 (8)°. The C6/O1/O2/C7 and C9/O4/O3/C10 mean planes form dihedral angles of 5.83(0.09)° and 14.86(0.10) °, respectively, with the mean plane through N1/N2/C2/C1/C3/C4/C5/N3, whereas the O8/C22/O7/C23 and C18/O5/O6/C17 mean planes form dihedral angles of 6.20(0.12) ° and 5.12(0.13) °, respectively, with the N5/N6/C14/C15/C16/C20/C21/N7 mean plane. In the crystal structure (Fig.2), molecules are linked by intermolecular N—H···O and N—H···N hydrogen bonds (Table 1) to form chains running parallel to the *b* axis.

### Experimental

A solution of ethyl chlorocarbonate (2.90 g, 12 mmol) in THF (30 ml) was slowly added dropwise to a mixture of *tert*-butyl 3-aminopyrrolo[3,4-*c*]pyrazole-5(1*H*,4*H*,6*H*)-carboxylate (3.3 g, 11 mmol) and DIEA (8.54 g, 66 mmol) in THF (60 ml) at 0~5 °C. The reaction was kept at the same temperature for 2 h, allowed to reach r.t., and stirred overnight. The obtained mixture was evaporated to dryness and the resulting residue extracted with AcOEt and water. The organic layer was separated, dried over sodium sulfate, and evaporated to dryness. The residue was purified by flash chromatography to give 1.8 g (55%) of the title compound as a white solid. Colourless block crystals suitable for X-ray diffraction were obtained in 2 days by slow evaporation of a methanol solution.

### Refinement

The hydrogen atoms could have been discerned in the difference Fourier map, nevertheless all H atoms were placed at calculated positions and refined as riding, with C—H = 0.96-0.97 Å, N—H = 0.86-0.90 Å, and with *U*_iso_(H) = 1.2 *U*_eq_(C, N) or 1.5 *U*_eq_(C) for methyl H atoms.

### Crystal data

**Table:**

C_13_H_20_N_4_O_4_	*Z* = 4
*M**_r_* = 296.33	*F*(000) = 632
Triclinic, *P*1	*D*_x_ = 1.343 Mg m^−^^3^
Hall symbol: -P 1	Mo *K*α radiation, λ = 0.71073 Å
*a* = 10.772 (3) Å	Cell parameters from 2737 reflections
*b* = 12.180 (4) Å	θ = 2.6–28.0°
*c* = 12.986 (4) Å	µ = 0.10 mm^−^^1^
α = 70.845 (5)°	*T* = 296 K
β = 65.875 (4)°	Block, colourless
γ = 85.821 (5)°	0.06 × 0.05 × 0.04 mm
*V* = 1465.2 (7) Å^3^	

### Data collection

**Table:**

Bruker APEXII CCD diffractometer	5102 independent reflections
Radiation source: fine-focus sealed tube	3915 reflections with *I* > 2σ(*I*)
graphite	*R*_int_ = 0.026
φ and ω scans	θ_max_ = 25.0°, θ_min_ = 1.8°
Absorption correction: multi-scan (*SADABS*; Bruker, 2008)	*h* = −12→10
*T*_min_ = 0.994, *T*_max_ = 0.996	*k* = −12→14
7444 measured reflections	*l* = −15→10

### Refinement

**Table:**

Refinement on *F*^2^	Primary atom site location: structure-invariant direct methods
Least-squares matrix: full	Secondary atom site location: difference Fourier map
*R*[*F*^2^ > 2σ(*F*^2^)] = 0.047	Hydrogen site location: inferred from neighbouring sites
*wR*(*F*^2^) = 0.128	H-atom parameters constrained
*S* = 1.03	*w* = 1/[σ^2^(*F*_o_^2^) + (0.0632*P*)^2^ + 0.4323*P*] where *P* = (*F*_o_^2^ + 2*F*_c_^2^)/3
5102 reflections	(Δ/σ)_max_ < 0.001
387 parameters	Δρ_max_ = 0.26 e Å^−^^3^
0 restraints	Δρ_min_ = −0.32 e Å^−^^3^

### Special details

**Table:**

Geometry. All e.s.d.'s (except the e.s.d. in the dihedral angle between two l.s. planes) are estimated using the full covariance matrix. The cell e.s.d.'s are taken into account individually in the estimation of e.s.d.'s in distances, angles and torsion angles; correlations between e.s.d.'s in cell parameters are only used when they are defined by crystal symmetry. An approximate (isotropic) treatment of cell e.s.d.'s is used for estimating e.s.d.'s involving l.s. planes.
Refinement. Refinement of *F*^2^ against ALL reflections. The weighted *R*-factor *wR* and goodness of fit *S* are based on *F*^2^, conventional *R*-factors *R* are based on *F*, with *F* set to zero for negative *F*^2^. The threshold expression of *F*^2^ > σ(*F*^2^) is used only for calculating *R*-factors(gt) *etc*. and is not relevant to the choice of reflections for refinement. *R*-factors based on *F*^2^ are statistically about twice as large as those based on *F*, and *R*- factors based on ALL data will be even larger.

### Fractional atomic coordinates and isotropic or equivalent isotropic displacement parameters (Å^2^)

**Table:**

	*x*	*y*	*z*	*U*_iso_*/*U*_eq_	
O1	0.26829 (15)	0.40073 (13)	0.06447 (14)	0.0348 (4)	
O2	0.15420 (17)	0.22918 (13)	0.11483 (14)	0.0383 (4)	
O3	0.18836 (13)	0.80172 (12)	0.11637 (12)	0.0248 (3)	
O4	−0.02679 (14)	0.86224 (13)	0.15737 (14)	0.0303 (4)	
O5	0.28280 (14)	1.06702 (13)	0.45631 (14)	0.0333 (4)	
O6	0.39625 (15)	1.23659 (13)	0.41233 (14)	0.0348 (4)	
O7	0.33735 (13)	0.66947 (12)	0.40945 (12)	0.0263 (3)	
O8	0.54850 (14)	0.63169 (12)	0.29063 (12)	0.0264 (3)	
N1	0.04383 (17)	0.39313 (15)	0.13325 (15)	0.0266 (4)	
N2	−0.08554 (17)	0.34058 (15)	0.17464 (16)	0.0278 (4)	
N3	0.03062 (16)	0.69338 (14)	0.12056 (15)	0.0252 (4)	
N4	−0.30512 (18)	0.40713 (16)	0.22648 (17)	0.0334 (5)	
H4"	−0.3627	0.4607	0.2482	0.050*	
H4'	−0.3438	0.3339	0.2641	0.050*	
N5	0.50579 (16)	1.08232 (14)	0.36264 (15)	0.0240 (4)	
N6	0.63493 (17)	1.14050 (15)	0.30111 (15)	0.0259 (4)	
N7	0.51226 (16)	0.79033 (14)	0.34994 (15)	0.0238 (4)	
N8	0.85181 (18)	1.08823 (16)	0.19749 (17)	0.0363 (5)	
H8'	0.8873	1.1559	0.1818	0.044*	
H8"	0.9026	1.0366	0.1726	0.044*	
C1	0.0358 (2)	0.50843 (17)	0.12258 (17)	0.0240 (5)	
C2	−0.1695 (2)	0.42522 (18)	0.18909 (18)	0.0265 (5)	
C3	−0.0945 (2)	0.53222 (18)	0.15632 (18)	0.0239 (5)	
C4	−0.1110 (2)	0.65547 (17)	0.15288 (19)	0.0252 (5)	
H4D	−0.1435	0.6998	0.0929	0.030*	
H4E	−0.1721	0.6611	0.2300	0.030*	
C5	0.1312 (2)	0.60819 (17)	0.08967 (18)	0.0247 (5)	
H5A	0.1848	0.5907	0.1369	0.030*	
H5B	0.1914	0.6337	0.0054	0.030*	
C6	0.1580 (2)	0.33069 (19)	0.10486 (18)	0.0289 (5)	
C7	0.3986 (2)	0.3522 (2)	0.0194 (2)	0.0403 (6)	
H7A	0.4667	0.4144	−0.0399	0.048*	
H7B	0.3917	0.2983	−0.0191	0.048*	
C8	0.4422 (3)	0.2901 (3)	0.1178 (2)	0.0500 (7)	
H8D	0.4388	0.3408	0.1619	0.075*	
H8E	0.5337	0.2679	0.0845	0.075*	
H8F	0.3822	0.2218	0.1703	0.075*	
C9	0.0578 (2)	0.79265 (17)	0.13261 (18)	0.0233 (5)	
C10	0.24457 (19)	0.90541 (17)	0.12164 (18)	0.0230 (5)	
C11	0.3866 (2)	0.87316 (18)	0.10925 (19)	0.0286 (5)	
H11A	0.3815	0.8011	0.1707	0.043*	
H11B	0.4315	0.9335	0.1168	0.043*	
H11C	0.4371	0.8643	0.0324	0.043*	
C12	0.1652 (2)	0.9233 (2)	0.24050 (18)	0.0303 (5)	
H12A	0.0756	0.9455	0.2468	0.046*	
H12B	0.2116	0.9837	0.2466	0.046*	
H12C	0.1577	0.8522	0.3038	0.046*	
C13	0.2481 (2)	1.00881 (18)	0.01703 (18)	0.0279 (5)	
H13A	0.2999	0.9927	−0.0559	0.042*	
H13B	0.2899	1.0760	0.0166	0.042*	
H13C	0.1567	1.0235	0.0239	0.042*	
C14	0.7175 (2)	1.06333 (17)	0.26074 (18)	0.0242 (5)	
C15	0.6416 (2)	0.95619 (17)	0.29503 (17)	0.0223 (4)	
C16	0.5126 (2)	0.97181 (17)	0.35743 (17)	0.0226 (4)	
C17	0.3930 (2)	1.13870 (19)	0.41168 (18)	0.0272 (5)	
C18	0.1527 (2)	1.1110 (2)	0.5106 (2)	0.0393 (6)	
H18A	0.1624	1.1630	0.5500	0.047*	
H18B	0.0880	1.0467	0.5704	0.047*	
C19	0.0999 (3)	1.1747 (3)	0.4194 (2)	0.0500 (7)	
H19A	0.1563	1.2452	0.3675	0.075*	
H19B	0.0080	1.1935	0.4585	0.075*	
H19C	0.1014	1.1266	0.3734	0.075*	
C20	0.4166 (2)	0.86791 (17)	0.40609 (18)	0.0245 (5)	
H20A	0.3799	0.8369	0.4926	0.029*	
H20B	0.3426	0.8839	0.3802	0.029*	
C21	0.65594 (19)	0.83642 (17)	0.28762 (18)	0.0227 (4)	
H21A	0.6951	0.8374	0.2055	0.027*	
H21B	0.7108	0.7922	0.3283	0.027*	
C22	0.4724 (2)	0.69102 (17)	0.34557 (17)	0.0223 (4)	
C23	0.2682 (2)	0.55632 (18)	0.43977 (19)	0.0269 (5)	
C24	0.3325 (2)	0.45964 (18)	0.5035 (2)	0.0318 (5)	
H24A	0.4243	0.4547	0.4499	0.048*	
H24B	0.2809	0.3872	0.5303	0.048*	
H24C	0.3335	0.4753	0.5710	0.048*	
C25	0.2688 (2)	0.5414 (2)	0.3285 (2)	0.0366 (6)	
H25A	0.2313	0.6073	0.2891	0.055*	
H25B	0.2148	0.4717	0.3500	0.055*	
H25C	0.3607	0.5358	0.2756	0.055*	
C26	0.1261 (2)	0.5688 (2)	0.5249 (2)	0.0355 (6)	
H26A	0.1310	0.5911	0.5876	0.053*	
H26B	0.0732	0.4958	0.5588	0.053*	
H26C	0.0837	0.6274	0.4825	0.053*	

### Atomic displacement parameters (Å^2^)

**Table:**

	*U*^11^	*U*^22^	*U*^33^	*U*^12^	*U*^13^	*U*^23^
O1	0.0307 (8)	0.0283 (9)	0.0448 (9)	0.0079 (7)	−0.0123 (7)	−0.0166 (7)
O2	0.0497 (10)	0.0235 (9)	0.0474 (10)	0.0099 (7)	−0.0223 (8)	−0.0171 (7)
O3	0.0228 (7)	0.0174 (7)	0.0364 (8)	0.0017 (6)	−0.0129 (6)	−0.0105 (6)
O4	0.0246 (8)	0.0212 (8)	0.0471 (9)	0.0035 (6)	−0.0139 (7)	−0.0151 (7)
O5	0.0252 (8)	0.0301 (9)	0.0415 (9)	0.0043 (7)	−0.0074 (7)	−0.0165 (7)
O6	0.0394 (9)	0.0243 (9)	0.0429 (9)	0.0074 (7)	−0.0140 (7)	−0.0184 (7)
O7	0.0257 (8)	0.0195 (8)	0.0348 (8)	−0.0016 (6)	−0.0130 (6)	−0.0089 (6)
O8	0.0308 (8)	0.0185 (8)	0.0308 (8)	0.0028 (6)	−0.0126 (6)	−0.0094 (6)
N1	0.0303 (9)	0.0179 (9)	0.0328 (10)	0.0031 (7)	−0.0125 (8)	−0.0106 (8)
N2	0.0319 (10)	0.0197 (9)	0.0331 (10)	0.0011 (8)	−0.0137 (8)	−0.0096 (8)
N3	0.0234 (9)	0.0187 (9)	0.0354 (10)	0.0032 (7)	−0.0120 (8)	−0.0115 (8)
N4	0.0298 (10)	0.0221 (10)	0.0455 (11)	−0.0031 (8)	−0.0124 (9)	−0.0103 (9)
N5	0.0252 (9)	0.0170 (9)	0.0291 (9)	0.0010 (7)	−0.0101 (7)	−0.0081 (7)
N6	0.0258 (9)	0.0191 (9)	0.0313 (10)	0.0001 (7)	−0.0100 (8)	−0.0083 (8)
N7	0.0241 (9)	0.0186 (9)	0.0309 (9)	0.0018 (7)	−0.0117 (7)	−0.0103 (7)
N8	0.0266 (10)	0.0200 (10)	0.0557 (13)	−0.0013 (8)	−0.0077 (9)	−0.0156 (9)
C1	0.0319 (11)	0.0191 (11)	0.0244 (10)	0.0021 (9)	−0.0134 (9)	−0.0088 (9)
C2	0.0340 (12)	0.0213 (11)	0.0256 (11)	−0.0009 (9)	−0.0134 (9)	−0.0070 (9)
C3	0.0274 (11)	0.0205 (11)	0.0269 (11)	0.0030 (9)	−0.0128 (9)	−0.0096 (9)
C4	0.0264 (11)	0.0188 (11)	0.0325 (11)	0.0003 (8)	−0.0133 (9)	−0.0090 (9)
C5	0.0267 (11)	0.0209 (11)	0.0290 (11)	0.0040 (9)	−0.0116 (9)	−0.0113 (9)
C6	0.0391 (13)	0.0229 (12)	0.0278 (11)	0.0068 (10)	−0.0148 (10)	−0.0116 (9)
C7	0.0344 (13)	0.0409 (15)	0.0446 (14)	0.0137 (11)	−0.0116 (11)	−0.0210 (12)
C8	0.0479 (15)	0.0555 (18)	0.0545 (17)	0.0147 (13)	−0.0253 (13)	−0.0244 (14)
C9	0.0248 (10)	0.0175 (10)	0.0254 (10)	−0.0007 (9)	−0.0097 (9)	−0.0047 (8)
C10	0.0221 (10)	0.0180 (10)	0.0280 (11)	−0.0034 (8)	−0.0092 (8)	−0.0068 (8)
C11	0.0268 (11)	0.0234 (11)	0.0346 (12)	0.0006 (9)	−0.0130 (9)	−0.0077 (9)
C12	0.0285 (11)	0.0338 (13)	0.0294 (11)	−0.0005 (9)	−0.0108 (9)	−0.0118 (10)
C13	0.0295 (11)	0.0211 (11)	0.0305 (11)	0.0025 (9)	−0.0107 (9)	−0.0074 (9)
C14	0.0284 (11)	0.0176 (11)	0.0270 (11)	0.0006 (9)	−0.0122 (9)	−0.0064 (9)
C15	0.0257 (11)	0.0175 (10)	0.0236 (10)	0.0007 (8)	−0.0108 (9)	−0.0055 (8)
C16	0.0284 (11)	0.0180 (10)	0.0237 (10)	0.0017 (8)	−0.0131 (9)	−0.0064 (8)
C17	0.0314 (12)	0.0255 (12)	0.0273 (11)	0.0054 (9)	−0.0125 (9)	−0.0119 (9)
C18	0.0292 (12)	0.0452 (15)	0.0402 (13)	0.0077 (11)	−0.0044 (10)	−0.0234 (12)
C19	0.0383 (14)	0.0593 (18)	0.0593 (17)	0.0192 (13)	−0.0192 (13)	−0.0320 (15)
C20	0.0247 (10)	0.0210 (11)	0.0285 (11)	0.0015 (8)	−0.0103 (9)	−0.0096 (9)
C21	0.0246 (10)	0.0169 (10)	0.0267 (11)	0.0009 (8)	−0.0110 (9)	−0.0065 (8)
C22	0.0276 (11)	0.0186 (10)	0.0225 (10)	−0.0001 (9)	−0.0148 (9)	−0.0027 (8)
C23	0.0323 (11)	0.0185 (11)	0.0330 (11)	−0.0043 (9)	−0.0175 (10)	−0.0054 (9)
C24	0.0380 (12)	0.0220 (12)	0.0354 (12)	−0.0013 (10)	−0.0192 (10)	−0.0033 (10)
C25	0.0477 (14)	0.0295 (13)	0.0372 (13)	−0.0068 (11)	−0.0231 (11)	−0.0075 (10)
C26	0.0305 (12)	0.0313 (13)	0.0441 (13)	−0.0056 (10)	−0.0174 (10)	−0.0073 (11)

### Geometric parameters (Å, °)

**Table:**

O1—C6	1.327 (3)	C7—H7B	0.9700
O1—C7	1.447 (3)	C8—H8D	0.9600
O2—C6	1.202 (3)	C8—H8E	0.9600
O3—C9	1.341 (2)	C8—H8F	0.9600
O3—C10	1.473 (2)	C10—C11	1.507 (3)
O4—C9	1.216 (2)	C10—C13	1.508 (3)
O5—C17	1.328 (2)	C10—C12	1.509 (3)
O5—C18	1.442 (3)	C11—H11A	0.9600
O6—C17	1.198 (3)	C11—H11B	0.9600
O7—C22	1.341 (2)	C11—H11C	0.9600
O7—C23	1.471 (2)	C12—H12A	0.9600
O8—C22	1.216 (2)	C12—H12B	0.9600
N1—C1	1.365 (3)	C12—H12C	0.9600
N1—C6	1.379 (3)	C13—H13A	0.9600
N1—N2	1.390 (2)	C13—H13B	0.9600
N2—C2	1.330 (3)	C13—H13C	0.9600
N3—C9	1.336 (3)	C14—C15	1.430 (3)
N3—C5	1.471 (3)	C15—C16	1.329 (3)
N3—C4	1.472 (2)	C15—C21	1.486 (3)
N4—C2	1.347 (3)	C16—C20	1.484 (3)
N4—H4"	0.8999	C18—C19	1.486 (4)
N4—H4'	0.8999	C18—H18A	0.9700
N5—C16	1.365 (3)	C18—H18B	0.9700
N5—C17	1.373 (3)	C19—H19A	0.9600
N5—N6	1.393 (2)	C19—H19B	0.9600
N6—C14	1.330 (3)	C19—H19C	0.9600
N7—C22	1.339 (3)	C20—H20A	0.9700
N7—C21	1.473 (2)	C20—H20B	0.9700
N7—C20	1.477 (3)	C21—H21A	0.9700
N8—C14	1.338 (3)	C21—H21B	0.9700
N8—H8'	0.8600	C23—C24	1.504 (3)
N8—H8"	0.8600	C23—C26	1.511 (3)
C1—C3	1.329 (3)	C23—C25	1.512 (3)
C1—C5	1.482 (3)	C24—H24A	0.9600
C2—C3	1.429 (3)	C24—H24B	0.9600
C3—C4	1.487 (3)	C24—H24C	0.9600
C4—H4D	0.9700	C25—H25A	0.9600
C4—H4E	0.9700	C25—H25B	0.9600
C5—H5A	0.9700	C25—H25C	0.9600
C5—H5B	0.9700	C26—H26A	0.9600
C7—C8	1.494 (4)	C26—H26B	0.9600
C7—H7A	0.9700	C26—H26C	0.9600
			
C6—O1—C7	116.59 (18)	C10—C12—H12A	109.5
C9—O3—C10	120.85 (15)	C10—C12—H12B	109.5
C17—O5—C18	117.13 (18)	H12A—C12—H12B	109.5
C22—O7—C23	121.69 (16)	C10—C12—H12C	109.5
C1—N1—C6	128.99 (18)	H12A—C12—H12C	109.5
C1—N1—N2	110.17 (16)	H12B—C12—H12C	109.5
C6—N1—N2	120.78 (17)	C10—C13—H13A	109.5
C2—N2—N1	104.91 (16)	C10—C13—H13B	109.5
C9—N3—C5	124.64 (17)	H13A—C13—H13B	109.5
C9—N3—C4	120.16 (17)	C10—C13—H13C	109.5
C5—N3—C4	114.81 (15)	H13A—C13—H13C	109.5
C2—N4—H4"	121.4	H13B—C13—H13C	109.5
C2—N4—H4'	119.3	N6—C14—N8	122.08 (18)
H4"—N4—H4'	112.9	N6—C14—C15	110.37 (17)
C16—N5—C17	128.92 (17)	N8—C14—C15	127.55 (19)
C16—N5—N6	110.18 (16)	C16—C15—C14	106.07 (18)
C17—N5—N6	120.77 (16)	C16—C15—C21	111.02 (17)
C14—N6—N5	104.90 (16)	C14—C15—C21	142.89 (19)
C22—N7—C21	121.67 (17)	C15—C16—N5	108.47 (17)
C22—N7—C20	123.51 (16)	C15—C16—C20	114.67 (18)
C21—N7—C20	114.55 (15)	N5—C16—C20	136.80 (18)
C14—N8—H8'	120.0	O6—C17—O5	126.8 (2)
C14—N8—H8"	120.0	O6—C17—N5	124.59 (19)
H8'—N8—H8"	120.0	O5—C17—N5	108.57 (17)
C3—C1—N1	108.48 (18)	O5—C18—C19	110.85 (19)
C3—C1—C5	114.49 (19)	O5—C18—H18A	109.5
N1—C1—C5	136.95 (19)	C19—C18—H18A	109.5
N2—C2—N4	121.98 (19)	O5—C18—H18B	109.5
N2—C2—C3	110.42 (18)	C19—C18—H18B	109.5
N4—C2—C3	127.6 (2)	H18A—C18—H18B	108.1
C1—C3—C2	106.00 (19)	C18—C19—H19A	109.5
C1—C3—C4	111.15 (17)	C18—C19—H19B	109.5
C2—C3—C4	142.74 (19)	H19A—C19—H19B	109.5
N3—C4—C3	100.28 (16)	C18—C19—H19C	109.5
N3—C4—H4D	111.7	H19A—C19—H19C	109.5
C3—C4—H4D	111.7	H19B—C19—H19C	109.5
N3—C4—H4E	111.7	N7—C20—C16	98.60 (15)
C3—C4—H4E	111.7	N7—C20—H20A	112.0
H4D—C4—H4E	109.5	C16—C20—H20A	112.0
N3—C5—C1	98.69 (16)	N7—C20—H20B	112.0
N3—C5—H5A	112.0	C16—C20—H20B	112.0
C1—C5—H5A	112.0	H20A—C20—H20B	109.7
N3—C5—H5B	112.0	N7—C21—C15	100.59 (16)
C1—C5—H5B	112.0	N7—C21—H21A	111.7
H5A—C5—H5B	109.7	C15—C21—H21A	111.7
O2—C6—O1	127.0 (2)	N7—C21—H21B	111.7
O2—C6—N1	123.8 (2)	C15—C21—H21B	111.7
O1—C6—N1	109.18 (18)	H21A—C21—H21B	109.4
O1—C7—C8	111.1 (2)	O8—C22—N7	124.23 (18)
O1—C7—H7A	109.4	O8—C22—O7	126.38 (18)
C8—C7—H7A	109.4	N7—C22—O7	109.38 (18)
O1—C7—H7B	109.4	O7—C23—C24	110.13 (16)
C8—C7—H7B	109.4	O7—C23—C26	101.64 (17)
H7A—C7—H7B	108.0	C24—C23—C26	109.97 (18)
C7—C8—H8D	109.5	O7—C23—C25	109.88 (16)
C7—C8—H8E	109.5	C24—C23—C25	112.93 (19)
H8D—C8—H8E	109.5	C26—C23—C25	111.72 (18)
C7—C8—H8F	109.5	C23—C24—H24A	109.5
H8D—C8—H8F	109.5	C23—C24—H24B	109.5
H8E—C8—H8F	109.5	H24A—C24—H24B	109.5
O4—C9—N3	123.50 (18)	C23—C24—H24C	109.5
O4—C9—O3	126.06 (18)	H24A—C24—H24C	109.5
N3—C9—O3	110.43 (18)	H24B—C24—H24C	109.5
O3—C10—C11	102.22 (16)	C23—C25—H25A	109.5
O3—C10—C13	108.64 (16)	C23—C25—H25B	109.5
C11—C10—C13	111.19 (16)	H25A—C25—H25B	109.5
O3—C10—C12	111.02 (15)	C23—C25—H25C	109.5
C11—C10—C12	110.14 (18)	H25A—C25—H25C	109.5
C13—C10—C12	113.09 (18)	H25B—C25—H25C	109.5
C10—C11—H11A	109.5	C23—C26—H26A	109.5
C10—C11—H11B	109.5	C23—C26—H26B	109.5
H11A—C11—H11B	109.5	H26A—C26—H26B	109.5
C10—C11—H11C	109.5	C23—C26—H26C	109.5
H11A—C11—H11C	109.5	H26A—C26—H26C	109.5
H11B—C11—H11C	109.5	H26B—C26—H26C	109.5
			
C1—N1—N2—C2	−0.5 (2)	C9—O3—C10—C13	−67.0 (2)
C6—N1—N2—C2	−178.02 (18)	C9—O3—C10—C12	58.0 (2)
C16—N5—N6—C14	−0.5 (2)	N5—N6—C14—N8	−179.34 (19)
C17—N5—N6—C14	−176.67 (18)	N5—N6—C14—C15	0.6 (2)
C6—N1—C1—C3	177.6 (2)	N6—C14—C15—C16	−0.5 (2)
N2—N1—C1—C3	0.4 (2)	N8—C14—C15—C16	179.4 (2)
C6—N1—C1—C5	−5.9 (4)	N6—C14—C15—C21	−179.0 (3)
N2—N1—C1—C5	176.8 (2)	N8—C14—C15—C21	0.9 (4)
N1—N2—C2—N4	178.65 (18)	C14—C15—C16—N5	0.2 (2)
N1—N2—C2—C3	0.4 (2)	C21—C15—C16—N5	179.20 (16)
N1—C1—C3—C2	−0.1 (2)	C14—C15—C16—C20	−177.62 (17)
C5—C1—C3—C2	−177.45 (17)	C21—C15—C16—C20	1.4 (3)
N1—C1—C3—C4	177.00 (17)	C17—N5—C16—C15	175.96 (19)
C5—C1—C3—C4	−0.4 (3)	N6—N5—C16—C15	0.2 (2)
N2—C2—C3—C1	−0.2 (2)	C17—N5—C16—C20	−7.0 (4)
N4—C2—C3—C1	−178.3 (2)	N6—N5—C16—C20	177.3 (2)
N2—C2—C3—C4	−175.7 (3)	C18—O5—C17—O6	0.6 (3)
N4—C2—C3—C4	6.1 (4)	C18—O5—C17—N5	−179.03 (17)
C9—N3—C4—C3	−165.54 (18)	C16—N5—C17—O6	−180.0 (2)
C5—N3—C4—C3	7.5 (2)	N6—N5—C17—O6	−4.6 (3)
C1—C3—C4—N3	−4.2 (2)	C16—N5—C17—O5	−0.4 (3)
C2—C3—C4—N3	171.2 (3)	N6—N5—C17—O5	175.03 (16)
C9—N3—C5—C1	165.14 (19)	C17—O5—C18—C19	86.1 (3)
C4—N3—C5—C1	−7.6 (2)	C22—N7—C20—C16	−166.46 (18)
C3—C1—C5—N3	4.7 (2)	C21—N7—C20—C16	7.7 (2)
N1—C1—C5—N3	−171.7 (2)	C15—C16—C20—N7	−5.4 (2)
C7—O1—C6—O2	4.9 (3)	N5—C16—C20—N7	177.7 (2)
C7—O1—C6—N1	−174.19 (18)	C22—N7—C21—C15	167.14 (18)
C1—N1—C6—O2	−178.0 (2)	C20—N7—C21—C15	−7.1 (2)
N2—N1—C6—O2	−0.9 (3)	C16—C15—C21—N7	3.3 (2)
C1—N1—C6—O1	1.1 (3)	C14—C15—C21—N7	−178.2 (3)
N2—N1—C6—O1	178.17 (16)	C21—N7—C22—O8	−0.6 (3)
C6—O1—C7—C8	−86.4 (2)	C20—N7—C22—O8	173.13 (19)
C5—N3—C9—O4	179.44 (19)	C21—N7—C22—O7	−179.94 (16)
C4—N3—C9—O4	−8.2 (3)	C20—N7—C22—O7	−6.2 (3)
C5—N3—C9—O3	−2.0 (3)	C23—O7—C22—O8	12.3 (3)
C4—N3—C9—O3	170.39 (16)	C23—O7—C22—N7	−168.41 (16)
C10—O3—C9—O4	−3.2 (3)	C22—O7—C23—C24	55.5 (2)
C10—O3—C9—N3	178.21 (16)	C22—O7—C23—C26	172.01 (17)
C9—O3—C10—C11	175.42 (17)	C22—O7—C23—C25	−69.6 (2)

### Hydrogen-bond geometry (Å, °)

**Table:**

*D*—H···*A*	*D*—H	H···*A*	*D*···*A*	*D*—H···*A*
N8—H8"···O4^i^	0.86	2.25	3.069 (3)	160
N8—H8'···N2^ii^	0.86	2.26	3.087 (3)	163
N4—H4'···N6^iii^	0.90	2.25	3.104 (3)	157
N4—H4"···O8^iv^	0.90	2.36	3.233 (3)	163

Symmetry codes: (i) *x*+1, *y*, *z*; (ii) *x*+1, *y*+1, *z*; (iii) *x*−1, *y*−1, *z*; (iv) *x*−1, *y*, *z*.
